# Absence of Association between Preoperative Estimated Glomerular Filtration Rates and Postoperative Outcomes following Elective Gastrointestinal Surgeries: A Prospective Cohort Study

**DOI:** 10.1155/2018/5710641

**Published:** 2018-03-06

**Authors:** Sivesh K. Kamarajah, Behrad Barmayehvar, Mustafa Sowida, Amirul Adlan, Christina Reihill, Parvez Ellahee

**Affiliations:** ^1^College of Medical and Dental Sciences, University of Birmingham, Birmingham, UK; ^2^Pre-Operative Assessment Unit, Queen Elizabeth Hospital Birmingham, Birmingham, UK

## Abstract

**Background:**

Preoperative risk stratification and optimising care of patients undergoing elective surgery are important to reduce the risk of postoperative outcomes. Renal dysfunction is becoming increasingly prevalent, but its impact on patients undergoing elective gastrointestinal surgery is unknown although much evidence is available for cardiac surgery. This study aimed to investigate the impact of preoperative estimated glomerular filtration rate (eGFR) and postoperative outcomes in patients undergoing elective gastrointestinal surgeries.

**Methods:**

This prospective study included consecutive adult patients undergoing elective gastrointestinal surgeries attending preassessment screening (PAS) clinics at the Queen Elizabeth Hospital Birmingham (QEHB) between July and August 2016. Primary outcome measure was 30-day overall complication rates and secondary outcomes were grade of complications, 30-day readmission rates, and postoperative care setting.

**Results:**

This study included 370 patients, of which 11% (41/370) had eGFR of <60 ml/min/1.73 m^2^. Patients with eGFR < 60 ml/min/1.73 m^2^ were more likely to have ASA grade 3/4 (*p* < 0.001) and >2 comorbidities (*p* < 0.001). Overall complication rates were 15% (54/370), with no significant difference in overall (*p*=0.644) and major complication rates (*p*=0.831) between both groups. In adjusted models, only surgery grade was predictive of overall complications. Preoperative eGFR did not impact on overall complications (HR: 0.89, 95% CI: 0.45–1.54; *p*=0.2).

**Conclusions:**

Preoperative eGFR does not appear to impact on postoperative complications in patients undergoing elective gastrointestinal surgeries, even when stratified by surgery grade. These findings will help preassessment clinics in risk stratification and optimisation of perioperative care of patients.

## 1. Introduction

With increasing ageing population, chronic kidney disease (CKD) is becoming increasingly prevalent [1, 2]. CKD is recognised as a risk factor for cardiovascular morbidity and mortality in the general population [3, 4]. A recent meta-analysis estimated the global rates of CKD to be 13%, with majority of patients having stage 3 CKD (i.e., eGFR 30–60%) [5, 6]. In parallel, there are rising numbers of patients undergoing elective surgery, and the impact of renal dysfunction on postoperative outcomes following surgery remains unclear. Current guidelines from the Association of Anaesthetists of Great Britain and Ireland (AAGBI) do not recommend perioperative risk stratification and management of patients with reduced estimated glomerular filtration rate (eGFR) undergoing surgery, since evidence in this area is limited.

Much data are available in the context of cardiac and vascular surgeries to demonstrate association between renal dysfunction and postoperative outcomes following cardiac and vascular surgeries. A recent systematic review and meta-analysis of 46 studies demonstrated a threefold increased risk of 30-day postoperative complications and acute kidney injury (AKI) in patients with eGFR <60 ml/min/1.73 m^2^ undergoing cardiac and vascular surgeries [7]. Furthermore, this review also demonstrated that patients with eGFR < 60 ml/min/1.73 m^2^ had increased risk of major cardiovascular events and all-cause mortality during long-term follow-up.

With strong, convincing evidence available for cardiac and vascular surgeries, this is however limited and nondefined for patients undergoing gastrointestinal (GI) surgeries. Recently, two studies evaluated the impact of preoperative renal dysfunction on mortality and cerebrovascular events in patients undergoing noncardiac surgeries [8, 9]. Although both studies included a large proportion of patients with gastrointestinal (GI) and hepatopancreatobiliary (HPB) surgery, these studies did not evaluate the impact of renal dysfunction in this subgroup of patients. To further define the role of preoperative renal dysfunction in GI and HPB surgery, this study sought to explore the relationship between preoperative eGFR levels and overall complication rates of patients undergoing elective GI and HPB surgeries. This study will provide important information for preoperative counselling on risk of postoperative complications of patients undergoing elective GI and HPB surgeries.

## 2. Methods

This prospective study identified consecutive adult (≥18 years) patients attending preassessment screening clinic (PAS) for GI and HPB surgeries from August 2016 to September 2016 at the Queen Elizabeth Hospital Birmingham (QEHB). Eligible procedures were those involving elective surgery on any part of the gastrointestinal tract or biliary tree, involving a hospital admission with an overnight stay. Patients undergoing day-case urological, gynaecological, vascular, or transplant procedures were excluded. This study was registered and approved by the local audit department. Patients' medical records were reviewed from the online patient notes, and the data were extracted on to a uniform database (Microsoft® Excel 2010) that was designed to include all relevant details pertinent to this study. Three coauthors were involved with data extraction (Sivesh K. Kamarajah, Behrad Barmayehvar, and Mustafa Sowida), and the data were then validated for accuracy by an independent fourth coauthor (Amirul Adlan).

### 2.1. Preassessment Clinics

At QEHB, all patients undergoing surgical procedures are referred by the surgeon to dedicated preassessment clinics according to surgery grade and comorbidities. Clinics are divided into low risk and high risk; numerically, these correspond to levels 1 and 2A and levels 2B and 3. Low-risk clinics are led and delivered by trained preassessment nurses, whereas high-risk clinics are led and delivered by more experienced nursing staff and consultant anaesthetists. Currently, there is no systematic way of allocating patients into these clinics as patients are risk-assessed individually by the surgeons.

### 2.2. Main Explanatory Variable

eGFR as a measure of renal function was the main explanatory variable. The eGFR was estimated using the Modification of Diet in Renal Disease-4 (MDRD-4) equation, which is one of the accepted and validated methods of evaluating kidney function used worldwide [10]. The unit used for the eGFR variable was ml/min/1.73 m^2^. Preoperative renal dysfunction was defined by using the cutoff eGFR value of ≤60 ml/min/1.73 m^2^, as it is the common cutoff that has been widely used and reported in the literature [7].

### 2.3. Explanatory Variables

Explanatory variables were collected to provide a risk‐adjusted estimate. Variables were predefined and selected based on clinical plausibility. To account for comorbidities, both the American Society of Anesthesiologists (ASA) fitness grade and the number of comorbidities (0 versus 1–2 versus >2) were measured. The ASA grade considers disease severity and is a reliable metric for the measurement of postoperative mortality and complications [11]. Grade of surgery is a category that indicates a combination of complexity and amount of tissue injury in the surgical procedure. Exact definitions used are similar to that used in a recent publication using definition from the European Surgical Outcomes Study, and these are provided in Supplementary Table 1 [9]. Surgical approach was defined as open, laparoscopic, or endoscopic/ultrasound (for minor surgical grade only).

### 2.4. Outcome Measures

The primary outcome in this study was the 30-day overall complication rates. Secondary outcome measures were grade of complications according to the Clavien-Dindo classification system [12], 30-day readmission rates, and postoperative care setting. According to the Clavien-Dindo classification system, complications were graded from grade I (any deviation from the normal postoperative course without the need for pharmacological treatment or surgical, endoscopic, and radiological interventions) to Grade V (death of patient following surgery). This allows comparison of the grades of complications in patients with and without renal dysfunction.

### 2.5. Statistical Analysis

Continuous variables were expressed as mean ± standard deviation or median (interquartile range) and analysed using *t*-test or Mann–Whitney test, where appropriate. Categorical variables were expressed as percentages and analysed using chi-square test or Fisher's exact test, where appropriate. Univariate logistic regression and multivariate logistic regression were used to determine the strength association between risk factors for postoperative overall complications. Clinically, important variables with *p* < 0.25 on univariate analysis were entered into multivariate analysis. The Hosmer-Lemeshow goodness-of-fit test and c-statistics were used to evaluate the model's performance and discriminative ability. Results are presented as hazard ratios (HRs) with 95 percent confidence interval (CI_95%_). In all analyses, a *p* value of <0.05 was maintained as statistically significant. Data analysis was undertaken using R Foundation Statistical Software (R 3.2.1, R Foundation for Statistical Computing, Vienna, Austria).

## 3. Results

### 3.1. Baseline Characteristics

**Figure 1:**
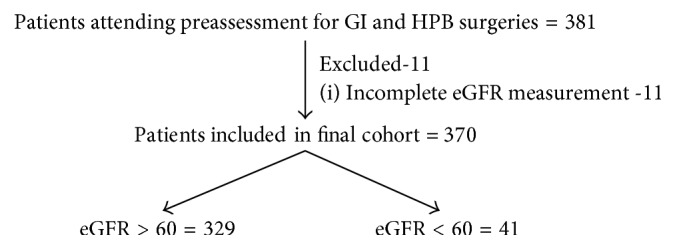
Flow diagram of included patients.

In this study, 370 patients undergoing elective gastrointestinal surgeries were included, of which 11% (41/370) had an eGFR ≤ 60 ml/min/1.73 m^2^, and the remaining 89% (329/370) had an eGFR > 60 ml/min/1.73 m^2^. A flow diagram of patients included in the study is presented in Figure 1. The baseline characteristics of included patients are presented in Table 1. Patients with eGFR ≤ 60 ml/min/1.73 m^2^ were significantly older than patients with eGFR > 60 ml/min/1.73 m^2^ (69 versus 54 years; *p* < 0.001). Patients with eGFR ≤ 60 ml/min/1.73 m^2^ also had significantly higher ASA grade 3/4 (36% versus 17%; *p* < 0.001) and >2 comorbidities (76% versus 37%; *p* < 0.001) than patients with eGFR > 60 ml/min/1.73 m^2^. Patients with renal dysfunction also had significantly higher rates of T2DM (32% versus 16%; *p*=0.025) and ischaemic heart disease (17% versus 5%; *p*=0.004). There was no significant difference in the surgical grade between the groups, although patients with eGFR ≤ 60 ml/min/1.73 m^2^ were more likely to have HPB surgery (63% versus 40%; *p*=0.008). There were equal rates of surgery for malignant indications and surgical approach between the two groups.

### 3.2. Indications for Surgery

The various indications for the included surgical cases are demonstrated in Table 2. The most common indication was malignancy (26%, 95/370), followed by hernia repair (23%, 85/370) and cholecystitis (19%, 70/370). In the group with eGFR ≤ 60 ml/min/1.73 m^2^, cholecystitis (27%) was the most common indication for surgery followed by malignancy (24%) and hernia repair (24%). In contrast, malignancy was the most common indication for surgery in patients with eGFR > 60 ml/min/1.73 m^2^.

### 3.3. Postoperative Outcomes

Postoperative outcomes for this cohort are presented in Table 3. Overall complication rates were 15% (54/370) across the whole cohort. The rates of complications were similar between two groups, with no significant difference in rates of 30-day overall complications (*p*=0.644). The 30-day readmission rates were 7% (23/324) in the group with eGFR > 60 ml/min/1.73 m^2^ and 5% (2/41) in the group with eGFR < 60 ml/min/1.73 m^2^. The rates of unplanned postoperative critical care admissions were 3% (11/326) in patients with eGFR > 60 ml/min/1.73 m^2^ and 2% (1/41) in patients with eGFR ≤ 60 ml/min/1.73 m^2^ (*p*=0.892). There were also no significant differences in the length of hospital stay between the two groups.

Binary multivariate logistic regression was used to identify factors predictive of overall complications in this cohort (Table 4). Univariate analysis identified age, sex, presence of type 2 diabetes mellitus (T2DM), major surgical grade, malignant, and open surgery to significantly predict postoperative complications. On multivariate regression, surgical grade (i.e., minor, intermediate, or major), presence of T2DM, and female sex were the predictive factor of overall complications. In adjusted models, preoperative eGFR did not show any impact on overall complications (HR: 0.89, 95% CI: 0.45–1.54; *p*=0.200). This nonsignificance remained even when the cohort was stratified by surgical grade.

Analyses were also repeated with eGFR cutoff as ≥45 ml/min/1.73 m^2^ (Supplementary Table 1). However, there were no significant differences in the rate of postoperative complications between patients with eGFR ≥45 ml/min/1.73 m^2^ and eGFR <45 ml/min/1.73 m^2^ (15% versus 0%; *p*=0.338). There were also no differences in the 30-day readmission rates and length of hospital stay (Supplementary Table 2).

## 4. Discussion

This prospective single-centre cohort study demonstrated no association between preoperative renal dysfunction and postoperative overall complication following elective GI and HPB surgeries. Instead, high surgical grade and the presence of T2DM were significant predictive factors of the 30-day overall complications. This demonstrates that risk stratification of patients according to eGFR may not be warranted from this study.

Although the association between chronic kidney disease and adverse postoperative outcomes has been described previously, most research have focused on high-risk patient groups where specific risk factors for renal dysfunction are more prevalent [7, 13]. Recently, a number of observational studies have examined preoperative eGFR and postoperative survival in noncardiac surgery [8, 9, 14–16]. The largest of these studies examined a retrospective cohort of over 250,000 patients undergoing noncardiac surgery from the American College of Surgeons National Surgical Quality Improvement Program (ACS-NSQUIP) data sets for 2005–2007 [14]. This study demonstrated that CKD and mineral bone disorders in patients undergoing general and vascular surgeries were independent predictors of early postoperative outcomes. However, this study included a broad range of procedures which may limit its applicability to GI and HPB surgeries. Furthermore, more recent evidence suggests that proteinuria may be a more important marker than eGFR for risk stratification of CKD [17, 18].

Despite these varying evidences demonstrating impact of eGFR, preassessment services do not consider eGFR as a risk factor during triage. Current literature is limited in the context of preassessment service in the UK and worldwide, advocating that a nurse-led and consultant/specialist-led PAS clinic is feasible but this remains nondefined considering the range of surgical specialities [19–21]. At QEHB, this two-tier clinic was recently introduced to allow assessment of patients by risk groups based on their comorbidities and ASA grade. This service allows risk assessment of patients for their comorbidities such as diabetes and cardiovascular disease prior to surgery, which may explain the nonsignificant findings in postoperative complications in patients with and without renal dysfunction.

The impact of renal dysfunction in normal physiology is often complex. Clinically, studies have demonstrated that patients with CKD are associated with higher rates of comorbidities such as T2DM, hypertension, hyperlipidemia, and cardiovascular disease, as is also seen in this study [4, 22]. Despite this, these diseases are not solely responsible for the adverse effects of CKD since strong associations remain after accounting for other factors on multivariable analysis from previous studies in patients undergoing cardiac surgeries [3, 4]. Furthermore, patients with CKD may have other risk factors such as elevated levels of inflammatory markers, homocystinemia, albuminuria, and elevated levels of uric acid which are often difficult to account for in studies [23, 24]. Alterations in these homeostatic mechanisms and others likely complicate normal recovery from abdominal surgery, specifically major surgeries.

One of the limitations of this study is that this is a single-centre study with a relatively small sample size. It was difficult to provide a formal sample size estimate as the complication rates of patients with and without preoperative renal dysfunction undergoing GI and HPB surgery is relatively unknown. However, this study provides early baseline data on 30-day overall complication rates between these two groups. Furthermore, this study only included patients undergoing elective procedures, and hence results may not be comparable to patients undergoing emergency GI and HPB surgery.

## 5. Conclusion

Preoperative eGFR level did not impact on postoperative overall complication rates even when stratified by surgical grades. Findings from this study suggests that the inclusion of patients with eGFR of <60 ml/min/1.73 m^2^ in high-risk PAS clinics for risk assessment based purely on eGFR alone for elective GI surgeries may not be warranted. Larger multicentre prospective studies should aim to better define the stage of impairment in renal function in the groups and assess the level of risk accordingly. This can potentially lead to a risk stratification scoring system to be used by clinicians in the preoperative care setting, which would help in identification, prioritisation, and optimising management of high-risk patients.

## Figures and Tables

**Table 1:** Baseline characteristics of patients.

	eGFR > 60 mL/min/1.73 m^2^ (*n*=329)	eGFR ≤ 60 mL/min/1.73 m^2^ (*n*=41)	*p* value
Age, years	53.6 (16.4)	69.4 (11.8)	<0.001
Sex, female	149 (45)	20 (49)	0.797
ASA grade			<0.001
Grade 1	39 (12)	1 (3)	
Grade 2	234 (71)	25 (63)	
Grade 3	55 (17)	11 (28)	
Grade 4	0 (0)	3 (8)	
Comorbidities			<0.001
0	55 (17)	1 (2)	
1-2	154 (47)	9 (22)	
>2	120 (37)	31 (76)	
Diabetes mellitus	53 (16)	13 (32)	0.025
Ischaemic heart disease	15 (5)	7 (17)	0.004
Congestive cardiac failure	5 (2)	0 (0)	0.938
Surgery grade			0.993
Minor	104 (32)	13 (32)	
Intermediate	142 (43)	18 (44)	
Major	83 (25)	10 (24)	
Surgical specialty			0.008
Upper GI	76 (23)	9 (22)	
Lower GI	120 (37)	6 (15)	
HPB	133 (40)	26 (63)	
Indication for surgery, malignant	82 (25)	10 (24)	1.000
Surgical approach			0.623
Endoscopic/ultrasound	102 (31)	15 (37)	
Laparoscopic	103 (31)	10 (24)	
Open	124 (37)	16 (39)	
Smoking status			0.686
Current	174 (53)	20 (49)	
Ex-smoker	77 (24)	8 (20)	
Never	73 (22)	13 (32)	
Unknown	3 (1)	0 (0)	
High-risk PAS clinics	91 (28)	14 (34)	0.493

Upper GI, upper gastrointestinal surgery; lower GI, lower gastrointestinal surgery; HPB, hepatopancreatobiliary surgery.

**Table 2:** Surgical indications.

Indications	eGFR > 60 mL/min/1.73 m^2^ (*n*=329)	eGFR ≤ 60 mL/min/1.73 m^2^ (*n*=41)
Malignant	85 (26)	10 (24)
Hernia	75 (23)	10 (24)
Cholecystitis	59 (18)	11 (27)
All other indications	25 (7)	2 (5)
Anal fistula	23 (6)	0 (0)
Diagnostic CLD	20 (6)	1 (2)
Haemorrhoids	15 (5)	0 (0)
Other liver or pancreatic disease	10 (3)	1 (2)
Inflammatory bowel disease	7 (2)	0 (0)
Gastro-oesophageal reflux	4 (1)	3 (7)
Faecal incontinence	3 (1)	2 (5)
Appendicitis	1 (0)	0 (0)
Diverticular disease	1 (0)	0 (0)
Pancreatitis	1 (0)	1 (2)

**Table 3:** Postoperative outcomes by eGFR.

Indications	eGFR > 60 mL/min/1.73 m^2^ (*n*=329)	eGFR ≤ 60 mL/min/1.73 m^2^ (*n*=41)	*p* value
Post-op complications			0.644
No	280 (85)	36 (88)	
Yes	49 (15)	5 (12)	
Complication grades			0.812
Grade 0	280 (85)	36 (88)	
Grade I	10 (3)	0 (0)	
Grade II	29 (9)	4 (10)	
Grade IIIA	2 (1)	0 (0)	
Grade IIIB	3 (1)	1 (2)	
Grade IV	4 (1)	0 (0)	
Grade V	1 (0)	0 (0)	
30-day readmission rate			0.596
No	301 (93)	39 (95)	
Yes	23 (7)	2 (5)	
Unplanned CCA			0.892
No	314 (96)	40 (98)	
Yes	11 (3)	1 (2)	
Postoperative setting			0.698
Ward	78 (24)	12 (29)	
Short stay	57 (14)	4 (10)	
ICU/HDU	54 (17)	8 (20)	
Ambulatory care	149 (45)	17 (42)	
Length of hospital stay, days	3.2 (2.1)	6.3 (4.2)	0.327

**Table 4:** Multivariate model for entire cohort in predicting postoperative complications.

	Univariate		Multivariate	
	OR (95% CI)	*p* value	OR (95% CI)	*p* value
Age	1.02 (1.00–1.04)	0.028	—	
Sex				
Male	Ref.		Ref.	
Female	2.59 (1.95–2.87)	0.025	1.85 (1.02–3.60)	0.045
ASA grade				
Grade 1	Ref.			
Grade 2	1.06 (0.42–3.24)	0.913		
Grade 3	2.06 (0.72–6.80)	0.198		
Grade 4	—	—		
Comorbidities				
0	Ref.			
1-2	2.14 (0.78, 7.54)	0.18		
>2	2.83 (1.04–9.93)	0.063		
T2DM				
No	Ref.		Ref.	
Yes	2.00 (1.00–3.83)	0.042	1.86 (1.24–4.03)	0.048
IHD				
No	Ref.			
Yes	0.92 (0.21–2.82)	0.896		
CCF				
No	Ref.			
Yes	4.01 (0.52–24.77)	0.133		
Surgical grade				
Minor	Ref.		Ref.	
Intermediate	2.29 (0.77–8.36)	0.161	2.65 (0.88–9.81)	0.103
Major	19.52 (7.39–67.56)	<0.001	21.38 (7.97–74.91)	<0.001
Surgical specialty				
Upper GI	Ref.			
Lower GI	0.81 (0.37–1.80)	0.592		
HBP	1.03 (0.51–2.20)	0.93		
Indication				
Benign	Ref.			
Malignant	4.24 (2.33–7.77)	<0.001		
Surgical approach				
Endoscopic/Ultrasound	Ref.			
Laparoscopic	5.00 (1.78–17.84)	0.005		
Open	8.71 (3.33–29.95)	<0.001		
PAS				
Low risk	Ref.			
High risk	5.33 (2.92–9.90)	<0.001		
eGFR				
>60	Ref.			
<60	0.79 (0.26, 1.96)	0.645		

Upper GI, upper gastrointestinal surgery; lower GI, lower gastrointestinal surgery; HPB, hepatopancreatobiliary surgery; PAS, preassessment service; eGFR, estimated glomerular filtration rate.

## Supplementary Materials

Supplementary Materials Supplementary Table 1: baseline characteristics of patients by eGFR. Supplementary Table 2: postoperative outcomes by eGFR.
