# Supplementary material for: Absence of Association between Preoperative Estimated Glomerular Filtration Rates and Postoperative Outcomes following Elective Gastrointestinal Surgeries: A Prospective Cohort Study
Source: Anesthesiol Res Pract. 2018 Mar 6;2018:5710641. doi: 10.1155/2018/5710641 (PMC5859863; doi:10.1155/2018/5710641)
Supplement: Supplementary Materials — Supplementary Table 1: baseline characteristics of patients by eGFR. Supplementary Table 2: postoperative outcomes by eGFR. [file 5710641.f1.docx]

|  | **eGFR >45** **ml/min/1.73m^2^,**  **n=359** | **eGFR <45** **ml/min/1.73m^2^,**  **n=11** | **p-value** |
| --- | --- | --- | --- |
| Age, years (SD) | 57 (15) | 71 (8) | 0.001 |
| Sex, Female | 163 (45) | 6 (55) | 0.77 |
| ASA Grade |  |  | <0.001 |
| Grade 1 | 40 (11) | 0 (0) |  |
| Grade 2 | 253 (71) | 6 (55) |  |
| Grade 3 | 64 (18) | 2 (18) |  |
| Grade 4 | 0 (0) | 3 (27) |  |
| Co-Morbidities |  |  | 0.003 |
| 0 | 56 (16) | 0 (0) |  |
| 1-2 | 162 (45) | 1 (9) |  |
| >2 | 141 (39) | 10 (91) |  |
| Diabetes Mellitus | 60 (17) | 6 (55) | 0.005 |
| Ischaemic Heart Disease | 20 (6) | 2 (18) | 0.274 |
| Congestive Cardiac Failure | 5 (1) | 0 (0) | 1 |
| Surgery Grade |  |  | 0.503 |
| Minor | 112 (31) | 5 (45) |  |
| Intermediate | 157 (44) | 3 (27) |  |
| Major | 90 (25) | 3 (27) |  |
| Surgical Specialty |  |  | 0.193 |
| Upper GI | 82 (23) | 3 (27) |  |
| Lower GI | 125 (35) | 1 (9) |  |
| HPB | 152 (42) | 7 (64) |  |
| Indication for Surgery, Malignant | 89 (25) | 3 (27) | 1 |
| Surgical Approach |  |  | 0.277 |
| Endoscopic/Ultrasound | 112 (31) | 5 (45) |  |
| Laparascopic | 112 (31) | 1 (9) |  |
| Open | 135 (38) | 5 (45) |  |
| Smoking Status |  |  | 0.487 |
| Current | 187 (52) | 7 (64) |  |
| Ex-Smoker | 82 (23) | 3 (27) |  |
| Never | 89 (25) | 1 (9) |  |
| High risk PAS clinics | 101 (28) | 4 (36) | 0.797 |

Supplementary Table 1 Baseline Characteristics of patients by eGFR

**Abbreviations - Upper Gastrointestinal Surgery (Upper GI), Lower Gastrointestinal Surgery (Lower GI), Hepatobiliary surgery (HPB)*

Supplementary Table 2 Post-operative outcomes by eGFR

|  | **eGFR >45** **ml/min/1.73m^2^,**  **n=359** | **eGFR <45** **ml/min/1.73m^2^,**  **n=11** | **p-value** |
| --- | --- | --- | --- |
| Post-op complications | 54 (15) | 0 (0) | 0.338 |
| Complication Grades |  |  | 0.38 |
| None | 305 (85) | 11 (100) |  |
| Minor (Grade I-II) | 43 (12) | 0 (0) |  |
| Major (Grade III-V) | 11 (3) | 0 (0) |  |
| 30-day readmission rate | 25 (7) | 0 (0) | 0.759 |
| Unplanned CCA | 13 (4) | 0 (0) | 1.000 |
| Post-operative Setting |  |  | 0.26 |
| Ward | 89 (25) | 1 (9) |  |
| Short Stay | 49 (14) | 2 (18) |  |
| ICU/HDU | 58 (16) | 4 (36) |  |
| Ambulatory care | 162 (45) | 4 (36) |  |
| Length of hospital Stay, days | 0 [0, 4] | 1 [0, 4] | 0.886 |
